# Book Reviews

**Published:** 1812-08

**Authors:** 


### CRITICAL ANALYSIS
### *OF RECENT PUBLICATIONS*
IN THE
### DIFFERENT BRANCHES OF PHYSIC, SURGERY, AND MEDICAL PHILOSOPHY.

### *Edinburgh Medical and Surgical Journal, No.* XXX.

### I. *Some Observations on Intus-Susception. By* Mr. Howship, Surgeon.

THIS is a case of not very usual occurrence, an intussusception of the rectum. A healthy female child, when four months old, was observed to have occasional sickness at the stomach. This state increased, until every thing that was swallowed was rejected soon afterwards.

"In this state, with very little variation, the infant remained for the space of three weeks, when a small quantity of blood was found upon the napkin, passed with a mucous slimy stool, attended with great great straining, restlessness, and apparently much pain. The parents, being alarmed at the appearance of blood, immediately procured medical advice.

"Gentle aperients were now ordered, but they could not be made to stay on the stomach. The child sickened, and the physic was thrown up. After various ineffectual trials, as it appeared that no relief was likely to be obtained by these means, a warm relaxing injection was ordered, and this was carefully thrown up; but the nurse said it all returned immediately without bringing any thing material with it. The following day a second injection was ordered, and given with no more effect than the first.

"Suspecting that there might have been some want of attention on the part of the nurse to explain this failure, the surgeon determined that he would himself ascertain, whether an enema might not be so injected as to operate more successfully. He was, however, not more fortunate than the nurse had been, for his attempts failed. Finding there was no chance of obtaining a passage by the bowels, little more was done ;the child was directed to be occasionally immersed in warm water, and this seemed to abate the heat and restlessness, but produced no permanent relief.

"The urgency of the tenesmus, the inability to keep any thing on the stomach, and the frequency in passing blood, all continued to increase rather than decline, attended with repeated attacks of most violent pain, for the space of six days, from the first bleeding, when the infant died, exhausted by her sufferings.

"On examination of the body, the following were the appearances. The viscera of the thorax were sound. In the abdomen, the stomach was apparently healthy. The small intestines had evidently suffered considerably from inflammation, and were, upon several parts of their surface, changing from a florid red to a dark and lurid color. The large intestines appeared, on the first glance, to be involved in confusion. There was, however, no difficulty in tracing the rectum upward from the pelvis, where it was very large, and felt, when pressed, as if it was full of a dense fleshy substance.

"Upon a more accurate examination of these parts, it appeared, that the lower portion of the colon, and the upper part of the rectum, had passed down into the lower extremity of the gut, producing a very extensive inflammation of the rectum, colon, and even the small intestines. The degree of stricture was very considerable, so that, when the external covering of the intestine rectum was divided, with a pair of scissars, the contained mass instantly sprung forward, occupying at least double its former space.

"The villous surface of the projecting extremity of the inverted intestine was of a dark and grumous color, being covered partly with blood, but principally with a ropy mucous matter."

When the disease can be ascertained during life, which the author presumes it may be by a careful examination, if in the rectum, he advises the cautious introduction of a bougie, " so large as to occupy the usual diameter of the

 intestine, intestine, and so curved and directed as to follow the natural turns of the gut, almost to its superior flexure."

II. *Case of Caries in the second cervical Vertebra, and consequent Fracture of its Dentiform Process. By* A. G. Kymell, Surgeon.—From the diseased state of the parts the spinal marrow became compressed, and the patient died paralytic in a few days. From the speedy termination of the disease, it does not appear that any thing in the way of medicine could be of service; the great object seems to have been the removal of compression by some mechanical contrivance.

III. *History of a Fever which prevailed in the Suburbs of Paisley; with an Account of the Utility of Blood-letting in imperfect Crises, slow Convalescence, and lingering Ailments. By* James Muir, Surgeon.—Thirteen families, residing in an elevated situation, in the suburbs to the south of the town of Paisley, in the months of January, February, and March, 1811, had thirty-two individuals seized with fever, one of whom, only, died.

"The fever generally commenced with shivering; in some there was excruciating pain of the back, and in others severe head-ache; the skin was dry; the tongue loaded or very red; the pulse quick, in many instances almost imperceptible, and in some, for many days, in a fluttering state. During the disease there came on much watchfulness, delirium, and seeming fatuity; in many cases there was pain in the abdomen, and in a few, painful micturition. The fœces were commonly brown, thin, and very fetid; in many instances, the bowels were loose, and in a few costive: both states were equally benefited by purgatives. In all cases there was great prostration of strength, and deep sickness. At the end of fifteen or sixteen days from confinement, signs of convalescence were observable, though there was seldom a well-marked crisis.

"The head-ache was relieved by opening the temporal artery or jugulur vein, and blistering the back of the neck. Cold affusion, even when employed on the first day of attack, did not stop, though it seemed to render the disease milder. Purgatives at first, and laxatives afterwards, afforded relief; but, at the commencement, emetics were entitled to the preference, as they sometimes arrested the progress of the disease at once, and invariably moderated, when they did not stop its course.

"One-fourth of the whole number relapsed, and the convalescence of many was extremely slow.

"Six of the thirty-two patients continued, after the 18th day, without any mitigation of the fever, which extended in some of these to the 4th and even to the 5th week. Two of them were much distressed with head-ache, one of whom had also for a few days slight pain at the breast; the other, who was at times in a very listless drowsy state, with flushed face, had severe pain in the lumbar region; the pulse in both seldom exceeded 100, was feeble, and sometimes fluttering. Three had slight cough, only one of whom

 had had any pain in the chest; but they all complained of a sense of weight or oppression at the pit of the stomach. Having failed to relieve the head-ache, and oppression of the præcordia, by purgatives, saline mixtures, and blisters, I had recourse to the lancet, in hopes of diminishing the head-ache; removing the load from the præcordia, and of giving a favorable direction to a crisis. The quantity of blood drawn did not exceed 11 ounces in any case; and in none was it necessary to repeat the operation, as the morbid affections of the head, the chest, and the belly, yielded immediately, or soon after the bleeding; the thirst abated, and the appetite returned.

"Five of the patients, after the usual period of convalescence, though free from fever, made almost no progress to recovery. One of them, while under the disease, was very delirious, and cried or screamed incessantly; he was deaf, passed numerous black stools, and had derived some relief from blistering. Although, after the fever, he took his food well for some weeks, he gained neither strength nor appearance; his skin was hard, his bowels loose, and his temper peevish: after losing a few ounces of blood, he was daily nourished by his food, the diarrhœa instantly ceased, and, from being extremely emaciated, he became soon plump and healthy. Another was freed from general debility, and cured of cough, by the detraction of eight ounces of blood. A third continued to languish without any particular complaint; but gradually attained health and strength, after a moderate bleeding. In the other two, who were young men, about fifteen years of age, the appetite and healthy appearance returned; but they continued stationary with respect to strength, and, though able to work a little, were soon fatigued. Blood-letting, to the extent of nine ounces, was followed by the same happy effects as in the three preceding cases, and one of these patients was almost instantly relieved from a strong palpitation at the heart, a symptom which I have observed in other cases as a sequel to fever. Of these five patients, only one was bled during the course of the fever."

This narrative sufficiently marks the occasional utility of blood-letting to relieve a tendency to inflammatory action in the system, and where much heat and irritation still remain, though in the advanced stage of fever. It must be allowed, however, that much nice discrimination and great caution will be requisite in the employment of this remedy in the period above stated. The history of the cases is given with distinctness, and the advantages arising from the abstraction of blood sufficiently marked, to substantiate its usefulness in these instances.

The following observations we cite, because the practice may be adopted without hazard to the patient.

"Recovery from fever may be greatly accelerated, and the danger of relapse lessened, by carefully swathing the body with flannel, in the manner recommended by Dr. White, and so extensively as well as successfully employed by Dr. Dewar for the cure of dysentery.

tery. Its good effects are the support and equable temperature it affords the body, and the preservation or restoration of the functions of the skin. This practice has been found beneficial in the continued fever of this country, by a gentleman who has had much experience of its efficacy; and this may be reasonably expected, when it is considered that, besides the advantages of repose, the external warmth of bed-clothes, by restoring the cutaneous discharge, does frequently more good in disease than all other curative means."

IV. *Medical Report for Nottingham. By* JAMES CLARKE, M.D.—We have long noticed Dr. Clarke as an indefatigable and intelligent observer of facts; but we are sorry to understand that this is intended to be the last of the Reports, which have occasionally emanated from his habit of noting occurrences, under the form of tabular registers.

We have not room to insert the Tables, which make the principal part of this Report, but shall present to our readers some cases which are not without considerable interest.

*Case of Hydrothorax successfully treated with Calomel and Squill.*

"Elizabeth Collingham, æt. 40. September 19th, 1809.—Complained of pain and oppression at chest; short cough; dyspnœa; can only lie whilst making considerable pressure on the chest; constant palpitation; pulse sharp but regular; starts in her sleep; passes but little urine; abdomen a little swelled; catamenia regular; bowels regular; has been ill six months.

Sumat pulv. jalap. comp. ℈jſs. cras mane.

℞. Hydrar. submuriat. gr. j.

Scillæ pulv. gr. iij.

Confect. sennæ q. s. ut f. pil. nocte maneque sumenda.

September 28th.—Has ptyalism, in other respects the same.

Omittr. medicament.

Sumat cochl. ij. larg. mixt. aromat. ter indies.

October 12th.—Reports much pain, particularly in the loins; bowels obstinately costive.

Sumat mixt. purg. com. ℥ij. 2da quaque hora, donec purgaverit. Rep. pil. hydrar. subm. et scillæ.

October 26th.—Dyspnœa removed, but little cough, free from pain, or palpitation of the heart; passes plenty of urine, but still complains of pain in the loins; bowels regular.

Persistat in usu pil.

November 9th —Considerably better.

Persistat in usu pil.

November 23d.—Reports very much troubled with the cough.

Persistat in usu pil.
Habeat decoct. lini. ad libitum.

December 14th.—Complains only of cough.

Persistat in usu pil. hydrar. submuriat.

January 25th, 1810.—Dismissed cured.

"The salutary effects of the treatment was soon evident in this case, after the mouth became affected; the symptoms gradually receded; receded; and the patient ceased to attend the hospital, having no further occasion for medical assistance."

*Two Cases of Hydrothorax successfully treated with Digitalis.*

"John Allan, soldier, æt. 34. December 5th, 1809.—Complained of much cough; pain at the chest; difficulty of breathing; pain in the bowels; furred tongue; quick and feeble pulse; legs swell towards night; bowels regular. Has been ill six months, and attributes his complaint to sleeping on the ground at night, during the campaign in Spain.

Sumat ij. pulv. emetic. vespere.
Sumat pulv. febrifug. ter indie.

December 7th.—Vomit operated freely, and has relieved the breathing; cough the same; pain at the chest and bowels undiminished; bowels open.

Persistat in usu pulv.
Applic. emplast. lyttæ sterno.

December 14th.—Pain moderated by the blister; cough and dyspnœa continue; he says he can feel the motion of water in the chest; bowels open and free from pain; urine in small quantity; cannot rest long, but on his left side; startings in his sleep; much dreaming; weight and tightness across the chest; palpitation; œdema of the feet; pulse very quick. Omit the powders.

℞. Digitalis pulv. gr. ſs.

Potassæ supertar. gr. ij. M. f. pulve. nocte maneque sumendus.

Sumat ʒjſs. pulv. jalap. comp. cras. sumend. mane.

December 21st.—Complains of faintness and lowness, which he attributes to his medicine; dypsnœa moderated; cough increased; other symptoms the same.

Contr. pulv. digital.

December 18th.—No amendment. Persistat in usu pulv. Rept. pulv. purg. cras mane.

January 4th, 1810.—Much the same. Persistat in usu med.

January 25th.—Can lie down with more ease, but is much disturbed in the night with violent coughing; still starts in his sleep, but seldom dreams; but little inconvenienced by palpitation; pulse small, feeble, and quick, but regular; urine small in quantity. Omit the powders.

℞. Digitalis pulv. gr. j.

Potassæ supertar. gr. ij. M. f. pulv. nocte maneque sumendus.

Sumat pulv. jalap. comp. ʒjſs. bis in septimana.

Habeat decoct. lini ad libitum.

February 3d.—Habeat in dies spirit. juniper comp. (gin punch) ℔ſs.

February 7th.—Continues to get better. Persistat in usu med.

February 19th.—Sumat mist. ferri comp. cochl. iij. larg. ter indie. Persistat in usu pulv.

March 7th.—Omitt. mixt. ferri comp. Persistat in usu pulv. digit. Applic. emplast. lyttæ scrobic. cordis. Sumat. mixt. mucilag. tussi urgenti.

March

March 19th.—Continues improving. Persistat in usu medicam.

March 29th.—Dismissed cured.

"The disease was in this instance the sequel of a typhus fever, arising from great fatigue, in the retreat of the army under Sir John Moore. The disease was completely removed by the practice employed; yet his constitution was so much impaired, that he was totally unfit for active service."

"Hezekiah Quirstam, waggoner, æt. 52. December 5th, 1809.—Complained of violent cough, with copious expectoration, the sputa streaked with blood; pain in the left shoulder; furred and tremulous tongue; much dyspnœa; feeble pulse; œdema of the legs towards night; extremities cold; bowels regular. Has been ill one month, and was afflicted with a similar disease last winter.

Sumat ij. pulv. emetic. vespere.

Sumat cochl. iij. larg. mixt. myrrh. comp. ter indies.

December 14th.—Vomit operated freely; continues much the same.

Persistat in usu mixt.

December 21st.—Dyspnœa undiminished; cannot rest on his back, but lies chiefly on his left side; cough very short and troublesome; is not troubled with unpleasant dreams or startings, but sleeps ill; urine in proper quantity; no œdema of the legs: bowels regular; extremities always cold; pulse regular.

℞. Digitalis pulv. gr. ſs.

Potassæ supertar. gr. ij. M. f. pulv. nocte maneque sumendus.

Sumat ℥jſs. pulv. jalap. comp. cras mane.

December 28th.—Dyspnœa moderated; cough the same.

Persistat in usu medicam.

January 11th.—In some respects better; bowels regular.

Persistat in usu medic.

February 8th.—Much better.

Persistat in usu medic.

February 22d.—Convalescent.

Persistat in usu med.

March 22d.—Free from complaint.—Dismissed.

"In this case the disease may be considered more as the sequel of pneumonia, than idiopathic hydrothorax; the man was restored to perfect health."

The following case of intestinal worms, we cite both for its evidence of the efficacy of turpentine, and from the derangement occasioned by the vermes assuming the character of pulmonary consumption.

"Sarah High, ætat. 27, Decem. 5, 1809, complained of violent pain in the bowels, much thirst, head-ach, fevered, furred tongue, much cough, bowels regular, catamenia regular. Has been ill two months.

Sumat. ℥j. ol ricini opt. statim.

Sumat. pulv. febrif. ter in die,

December

December 7th. Castor oil operated freely; has but little diminution of pain; less head-ache; thirst and fever the same; cough more full, with copious expectoration. Persistat in usu pulv. febrif.

℞. Hydrar. submuriat.
Ipecacuan. āā gr. j.
Extract. hyoscyami gr. vj. M. f. pil ij. omni nocte sumendæ.

December 14th. No amendment.
Persistat in usu medic.

December 28th. Cough very troublesome; vision much affected by the hyoscyamus. Omitt. medic.
Sumat. ʒſs. pulv. acaciæ comp. ter indies.
Applic. emplast. lyttæ sterno.

January 4th, 1810. Pain moderated; cough no better.
Persistat in usu medic.

January 11th. Cough very severe.
Persistat in usu medic.

January 25th. Symptoms undiminished.
Persistat in usu medic.

February 1st. Cough still urgent; has epistaxis.

Omitt. medic.

Sumat. ℈j. pulv. jalap. comp. cras mane.

℞. Magnes sulphat ℥j.

Infus rosæ, ℥xvj. M. f. mixtura cujus sumat. cochl. iij. larg. ter indies.

February 8th. Purgative gave relief; epistaxis removed; cough moderated. Persistat in usu medic.

March 8th. Has continued much the same.
Persistat in usu medicam.

March 28th. Complains of much pain in the left clavicle.
Persistat in usu medic. Rept. pulv. purgans cras mane.

April 5th. Has passed an extraordinary quantity of small worms; much dyspnœa; short cough; and gnawing pain at stomach.
Omitt. medic.

℞. Pil. aloes cum myrrh.

Ferri carbonat.

Extract. gentian āā ʒj.

Ol. rutæ gutt. xij. M. f. pil. 36, quarum sumat iij. bis indies.

April 19th. Passes worms.
Persistat in usu medic.

May 3d. Continues to pass worms; much cough.
Omitt. medic. Sumat. cochl. j. larg. ol. Terebinth, essent. statim et cras mane.

May 7th. The turpentine blistered the mouth and throat; occasioned much nausea, head-ache, general distress, and fits; the cough increased; much thirst; has passed many dead worms.
Sumat. ol. ricini opt. ℥ſs altera quaque mane.

May 18th. Continues to pass worms.
Sumat. cochl. iij. larj. mixt. salin. camph. ter indies.

May

May 31st. Has passed a considerable quantity of worms, and feels completely relieved; free from complaint. Dismissed.

"This woman had often been a patient of the hospital, for symptoms similar to what she related on her admission this time. Her general form was that observed in those disposed to consumption, and her complaints had so much general resemblance to that disease, that she was marked as one who would, sooner or later, fall a sacrifice to it. On the fifth of April she first mentions having passed worms: indeed, before this, they were not even suspected; and on the 3d of May she took about half an ounce of the essential oil of turpentine, mixed with honey: the effect was so violent, that her relatives came in great distress, with the remains of the bottle to be examined, suspecting that she was poisoned by some mistake in the medicine. It is proper to remark, that she has been troubled with fits from her childhood. It was not necessary to repeat the remedy; one dose proved sufficiently powerful; nor has she experienced any return of the symptoms until this month (May, 1811), when she again complained of gnawing pain at her stomach, short dry cough, &c. The turpentine was again exhibited, with the good effect of discharging worms, and removing her complaints, without inducing any violent effects."

The fluctuations of modes and fashions is felt as much in medical practice, as in the opinions of mankind on any other subject; and in these mutations we do not unfrequently observe an indication of return to old habits. In the time of Sydenham, and after him of Huxham, it was the practice (at least of these celebrated physicians) to keep registers of particular seasons, containing the states of the atmospherical changes, and the diseases occurring with such changes, under the denomination of the epidemic constitution of particular periods. We are by no means dissatisfied with observing Dr. Clarke to have adopted this good old fashion, and even under the old term.

The Epidemic Constitution from April 1810, to March 1811, as given by Dr. Clarke, may serve as a model for this kind of Register: we think, however, that the atmospherical changes might be more distinctly noted, for the great object is to ascertain, if possible, how diseases and their increase or subsidence, are connected with, and influenced by, those changes.

"April.—Rain fell in the night of the 2d, 3d, 8th, 17th, and 18th. The weather of this month was unusually fine. The prevailing diseases were pneumonia and catarrh; scarlatina, with and without ulcerations of the tonsils; and cynanche tonsillaris without eruption.

"May.—Rain fell in the night of the 1st, 7th, 8th, 20th, and 21st; snow in the night of the 4th, and ice on the ground an inch in thickness. The prevailing diseases of the month were synochus or common fever, particularly among children; ophthalmia; and epidemic scarlatina.

" *Phthisis,* that dreadful and fatal enemy to the inhabitants of manufacturing towns, presented fifteen subjects in this month. A near view of the manners of the lower class of people will, in a great measure, remove any surprise that the actual number dying of this disease might excite.

" June.—Rain fell on the night of the 9th and 19th; a thunder-storm on the 26th. The prevailing disease of this month was *pneumonia; scarlatina* still showed itself. One case of *small-pox* was brought into the town, but prevented from spreading, care being taken to vaccinate all those persons exposed to its immediate influence.

" July.—Rain in the night of the 1st, 10th, 12th, and 13th; thunder on the 1st and 13th. Two more cases of small-pox were brought into the town, but the contagion did not spread.

" August.—The wind chiefly southerly this month; was unusually healthy.

" September.—Rain in the night of the 21st and 23d.

" *Scarlatina* was very prevalent, passing through whole families; it was attended with inflammation, and often ulceration, of the tonsils, but in many instances much inflammation was found in the tonsils, unattended by rash. The scarlatina, towards the end of this month, often terminated in inflammation of the submaxillary and parotid glands, which generally proceeded to suppuration. Œdema of the extremities, and often of the face, succeeded the fever, if purgatives had not been employed: it was only to be removed by a course of purgatives, which in no instance increased the debility.

*Synochus* and *synochus infantum* were also prevalent diseases of this month. One more case of small-pox was brought into the town; the security afforded by vaccination was undiminished; the disease did not extend to another individual.

" October.—Rain in the nights of the 16th, 17th, 18th, 19th, 20th, 21st, and 27th; snow on the 29th; the wind chiefly easterly. *Scarlatina* continued through this month with an increased violence, and proved often fatal; it was, in many instances, attended with cough. *Typhus* became very prevalent and very fatal; in many cases the termination was unexpectedly sudden. Towards the end of the month the disease began with vomiting and purging; the diarrhœa was not moderated by opiates; small doses of calomel and ipecacuanha was found the most effectual treatment. *Synochus* was unusually prevalent and severe.

" November.—Rain fell in the night of the 1st, 5th, 9th, 10th, 14th, 15th, 16th, 17th, 18th, 20th, and 29th; wind variable. *Typhus,* for a few days in the beginning of this month, was attended with pain in the chest and sides, but the prevalence of this disease, as well as of the *scarlet fever,* was much diminished.........

" On examining the body of a woman who died of hydrothorax, a calculus was found in the gall-bladder, the existence of which was not not suspected; it is brown, rough, light, and friable, but not easily fractured: its long diameter measured one inch, short diameter ¾ of an inch, and it weighed 64 grains. Dr. Lettsom mentions the case of a biliary calculus, discharged by the rectum after some years of painful suffering, which measured in length 2¼ inches, and in circumference 3½ inches, and weighing one ounce, two drams, and twenty-three grains. 'It had,' he says, 'the natural appearance of a brown flint stone, though of less ponderosity, in proportion to its magnitude.'*

"December.—Rain in the night of the 3d, 4th, 5th, 12th, 22d, 24th, 25th, 26th, and 27th; snow in the night of the 9th, and 30th; boisterous westerly winds. This month was generally healthy; a few sporadic cases of scarlatina and cynanche tonsillaris were met with.

"January, 1811.—Rain fell in the night of the 10th, 11th, and 31st; snow in the night of the 3d, 4th, 15th, 27th, and 30th; wind chiefly westerly, and often boisterous.

"*Pneumonia* and *catarrh* were the prevalent diseases of this month; *scarlatina*, *rubeola*, and *typhus*, were occasionally met with. Four cases of variola were brought into the town, but the disease did not spread to any other individuals.

"February.—Rain fell in the night of the 6th, 7th, and 23d; snow in the night of the 13th, and 15th; the wind mostly westerly. *Catarrhus*, *synochus*, and *typhus*, particularly among children, are the most prevaling diseases. *Pertussis* and *scarlatina* appeared sporadically. The *typhus* is attended with a troublesome diarrhœa. The disease has its origin in the extreme distress of the poor, who, not being able to purchase wholesome food, are obliged to subsist on what they can procure, to satisfy the call of hunger. Many hundreds of frame-work knitters are out of employ, and dependent, with their families, on parochial relief. So much distress was never before witnessed in the stocking manufacture. A riotous disposition is manifesting itself in the town and county, and, if relief be not afforded, the consequences must be dreadful. The last severe contagious typhus in Nottingham was in the season of great scarcity (1798). There is every reason to fear the same effect may be produced from the present distress of the laboring poor. It is generally admitted, that typhus is never more virulent and contagious, than when it appears among the impoverished manufacturers.

"March.—Rain fell in the night of the 5th, 6th, 7th, and 21st; the wind mostly westerly.

"The *typhus* fever extended its ravages during this month; the *catarrhal* fever assumed the typhoid character."

V. *Case of Scirrhous Stomach, and Stricture in the Colon, with Appearances on Dissection.* By JOHN HOLMES, M.D.—A man, 39 years of age, by occupation a fisherman, in the summer of 1809 suffered by dyspepsia, nausea, acid eructa-

* Memoirs of the Medical Society of London, vol. i. p. 373.

 tions, tions, flatulence, and frequent vomitings of a thin, watery, and sometimes hot and acid, fluid. A peculiar uneasiness in the region of the stomach also gave more distress than in common cases of dyspepsia. In Feb. 1810, all these symptoms were increased, aggravated by a vomiting of all food, and a constant pain in the epigastric region. Though he was considerably emaciated, his countenance was healthy, and his bowels regular. In April the disease rapidly increased—debility prevented him rising from his bed—there was a slight diarrhœa, and some blood was discharged per anum. The pain in the epigastric region became excruciating, extending at times over the whole abdomen, and to the shoulders and arms. On laying my hand over the epigastric, says Dr. Holmes, where the pain was most severe, *I have more than once perceived a gurgling noise, like that of a fluid forcibly driven through a narrow aperture, in that part of the abdomen beneath my hand.*

" *Dissection.* The skin, abdominal muscles, and peritonæum, being cut through, the viscera of the abdomen presented themselves in their usual situation. On examining the stomach, it was discovered that the coats of the greatest part of the lower half of that viscus were prodigiously thickened, and entirely in a scirrhous condition.

" Nearly that half of the circumference of the pylorus, which is a continuation of the lesser curvature of the stomach, was completely scirrhous; and, although the remaining part of the circumference of the pylorus seemed in its natural condition, yet the calibre of the passage from the stomach into the duodenum appeared to be much diminished. The cardia, and upper part of the stomach, were in a sound state. The intestines being further examined, a most remarkable stricture was found in the great arch of the colon. The situation of this stricture was so precisely in the centre of the great arch of the colon where it passes through the epigastric region, that it must have been in the immediate vicinity of the pylorus; hence it is obvious, that whether the pain which the deceased experienced so severely during his life-time in the epigastric region, originated from the stricture in the colon, or from the morbid condition of the lower part of the stomach and pylorus, it must by him have been constantly referred to the same seat. A considerable quantity of feculent matter was found to have accumulated to the right of the stricture in the colon, while the intestine on the left of the stricture was nearly empty. The coats of the colon at this part were so morbidly thickened and contracted, that the little finger could scarcely be pushed with freedom through the aperture which remained for the passage of the fœces.

" The mesentery also was in a very morbid state; small circular spots of a whitish appearance were interspersed over the greatest part of it: when these spots were compressed between the fingers, they they were found to be small hard bodies, seemingly of a cartilaginous structure. A considerable part of the peritonæum was in a very similar condition; small white or greyish spots were thickly interspersed in it, but they were not so hard or cartilaginous as those of the mesentery; they seemed to partake of the same morbid structure, though in a less advanced state. The liver, kidneys, and other viscera of the abdomen, were in a sound state."

VI. *Cases of Emphysema, cured by the use of Cold Applications. By* P. JOHNSON, Surgeon.—These two cases, as it should appear, of spontaneous Emphysema, were cured by the application of cold effusion, or the bath of sea water cold. The mode of treating these cases, and the cure effected by it, are both sufficiently singular to excite attention to the cold bath in similar instances.

VII. *Miscellaneous Communications. By* WM. SIMMONS, Surgeon.—The first of these gives some account of an anomaly in vaccination. This anomaly is an instance, in Mr. Simmons himself, of receiving the vaccine disease in its regular and efficient form, after having, many years before, passed through the small-pox.

2d. *On the utility of Diuretics in Ulcers situated on the lower Extremities.*—The production of an increased flow of urine, Mr. Simmons found a powerful auxiliary in the cure of these ulcers, accompanied, generally, with an œdema of the leg.

3d. *On the property of Arsenic in Cancer.*—This is on a subject of such importance, that we shall cite the whole of Mr. Simmons's observations.

"The property of arsenic, taken internally, of allaying the pain of cancer in the ulcerated stage, is already before the public. In the common cancer, and in that variety of cancer to which chimney-sweepers are incident, its effects are nearly similar. In both it improves the quality of the discharge from the ulcer, and alleviates the pain, without inducing stupor or disposition to sleep. Hence it may be considered an *antalgic* rather than an *anodyne* in such cases.

"In the year 1810 an elderly gentlewoman took the 'liquor arsenicalis' in a case of cancer of the right breast, twice a day for three months. Her sufferings were extreme before she entered on this course; soon, however, the arsenic gave her ease, and, with a few exceptions, kept her easy during the remainder of her life. These exceptions for the most part took place when the tubercles were about to exfoliate; after the exfoliation, the pain again subsided, the hollow granulated; the granulations were florid and healthy-looking, and the secretion from them purulent. By these means, she was kept tolerably easy, compared with what is usually suffered, as she gradually sunk; nor did she experience any of those unpleasant symptoms which are said to arise from the continued exhibition of arsenic.

"As

"As the pain in the common, and in the chimney-sweeper's or soot cancer, is alike alleviated by the internal exhibition of arsenic, may we thence infer the *identity* of the poison in these two diseases? If a different cause can produce the same morbid change of structure in a part, there is no difficulty in conceiving a similarity of function in the subsequent action of the part, however dissimilar the original cause might have been."

4. *On the property of Iron in Cancer.*—Two cases of cancer are related, in which the treatment was conducted according to the suggestions of Mr. Carmichael. In both instances there was a mitigation of the burning sensation spread over the ulcerated surface; but this was not permanent; and both cases terminated fatally.

5. *Case of Occult Cancer.*—There was nothing unusual in the operation. "On examination of the tumor after extirpation, *it appeared to consist of an homogeneous substance, of a texture so hard as to resound when scraped with the scalpel; but there were no white bands, or intersections of any kind.*"

6. *On the Liquor Ferri Alkalani* (Lond. Pharm. 1809) *in Scrofula.*—In swellings of conglobate glands, and in scrofulous ulceration, Mr. Simmons has exhibited this remedy extensively, and with more benefit than any other preparation of iron or cinchona. He prescribes it in the common dose, largely diluted with water, two or three times a day, according to the sensible effects, increasing or lessening the dose, so as to procure not more than two evacuations from the bowels in twenty-four hours. Besides its operation on the bowels, the kidneys are stimulated to an increased action, and a considerable flow of urine is often the consequence. To these properties of a diuretic, and an aperient, belong those of a tonic, since the appetite and general strength are materially improved by it.

VIII. *Observations on those Accidents commonly ascribed to the Wind of a Ball. By* John Spence, M. D.—In a former number of our Journal, we had occasion to notice a very ingenious paper, by Mr. Ellis, on the mode by which the supposed "Wind of a Ball" produces those extraordinary effects which have been attributed to it. Mr. Ellis accounted for the phenomenon by calling in the aid of electricity, and he supported his hypothesis by recounting many instances of somewhat similar effects produced by lightning. On the whole, from the spirit of philosophical investigation which ran through this paper, and the support it seemed to receive from analogy, and from apparent facts, we were disposed to allow him the credit of coming very near the real cause of this mysterious effect. But the plain sense and unaffected statement of the paper before us, by Dr. Spence, has very much much lessened what we were willing to concede to Mr. Ellis. On a subject of such considerable interest, and where it is so desirable to ascertain the true fact, it is, perhaps, best to let the parties speak for themselves.

"The sudden concussion or agitation of the contiguous atmosphere, by the rapid motion (says Dr. Spence) of such a small body as a cannon-ball, does not appear to be sufficient to account for the extent of injury often produced, and alleged to be from that cause only. In all the cases that have fallen under my treatment and observation, I think I have been able to account for the cause more satisfactorily to myself; and the numerous occurrences in every battle, of cannon-balls passing close to the body without doing injury, should, at least, induce us to some investigation, before leaving it to a supposed cause, which may lead to a careless treatment.

"There are many substances, comparatively light of themselves, on board ship, such as canvas, rope-yarns, part of the bedding, &c. which, when carried along with the velocity of a ball, or even driven but a short way with that force, are apt to do considerable injury by hitting the body, and, by occupying a large or small bulk, may, or may not, produce external mark of injury. I know an officer who had considerable pain in the loins for some time after a battle, supposed to be from the wind of a shot, as he was not conscious of any thing having hit him, and there was no external mark; but the flakes of a bed from the hammock-knittings being pretty thick on his coat, shewed to me that the pain was produced by something more dense than wind; and had it been the stomach that was hit, instead of the back, I think it very probable that officer would have been killed. It is well known that a smart blow on the stomach will produce faintness, and sometimes deprive a person of life, without producing any external mark, which often happens with boys or men fighting; and it has happened, that a person has been killed by the sudden push of a foil, which bursted the peritonæum, without causing any external mark. Surely neither of these will be imputed to the effects of electric matter. As to its often happening that the person is not conscious of having been struck by any thing when those injuries are received, it is not to be wondered at, when it is known, that sometimes a severe wound is inflicted, and the person not know it until it is pointed out to him, as he gets faint by loss of blood. I knew a man that was wounded with a flattened ball, full two inches in diameter, which lodged deep in the thigh; and when getting faint with the loss of blood, asked his officer for permission to go to the surgeon, as he was afraid he was wounded somewhere, for he was not able to stand. I have also known a musket-ball pass through a part of the body, and the person not be conscious of having been wounded, until he discovered the blood: and many instances I have known, of injuries about the face discovered after an action, which had not before been noticed. I have always imputed those little accidents to some light substance having been brought, with great velocity, in contact with the face: but in what way they occurred, occurred, whether from the wind of a shot, part of the ship's material, electric matter, or latent caloric developed, I believe it will be difficult for the individual to ascertain in time of action at sea. As to the negative circumstances which occur against the opinion of the wind of a shot, or even electricity, or latent caloric, being the cause of those accidents, the instances are very numerous of shot passing very close to the body without doing any injury. In those cases, I suppose, there is nothing carried along with the ball, for the jacket or trowsers, and even the waistcoat, is sometimes torn, without the skin being injured, and even buttons are torn from the clothes by a ball; but this cannot be wondered at when we know that a button may be struck off a coat, by a smart blow with the end of a ramrod, often without the person perceiving it, and no one will think of imputing this to the attractive power of electricity. I knew an instance of a shot which passed through both sides of the cabin of a frigate, and in its course struck a store-room key (a common sized door key), that was lying loose, with one half of it exposed behind a looking glass, at the side of the cabin. The key was bent considerably without being moved from its place, neither did the glass receive the slightest injury. A dollar has been known to have been broken in many pieces in the pocket, by a musket-ball, without the person receiving any injury.

"Why should not every cannon-ball that carries away a limb, or wounds the body, bring along with it its other baneful influence? If the cause is produced by the velocity, it cannot be supposed that hitting the body in passing can have impeded much of the velocity; and there are numerous wounds by cannon-balls, where no other part is injured but what has been in actual contact with the ball."

These observations, and the facts stated with them, certainly militate very much against Mr. Ellis's conclusion.

Dr. Spence does not confine his observations in this paper to "wind of a ball," but goes on to notice an intermediate accident, between the atmospherical action, and the absolute stroke of a ball, under the term of "*the brush of a ball.*"

The brush of a ball is that injury which arises when a ball has passed so near, as actually to touch the clothes, and neither tear them nor the skin. This often occasions an accident of great importance, producing numbness, swelling, inflammation, and sphacelus. But this, as secondary to the main object of investigation, we shall notice no farther at present.

IX. *Report of the Board of Health at New York, on the Yellow Fever at Perth-Amboy.*—This Report is prefaced by a letter from Dr. Chisholm, containing observations on its facts, which the doctor considers as affirmative of the infectious nature of the yellow fever.

The importance of the question respecting the infectious or non-infectious quality of the yellow fever, seems to require quire that we should lay the whole of this Report before our readers, as an authentic document of the existence of a fact we should be happy to see no longer disputable.

" *Board of Health, September* 16, 1811.—Present, the Honorable Dewitt Clinton, President.—*Aldermen,* Mesier, Carpenter, Fish, C. Pell, Cunningham, Buckmaster, J. Pell, Dickinson, Hoogland, Torrey.—*Doctors,* Miller, Bailey, and Douglass.

" Information having been given to the Board, that a dangerous, malignant, and infectious, fever now prevails at the city of Amboy, in New Jersey, it was resolved that it be recommended to his honor the mayor to issue his proclamation, interdicting all communication between the said city of Amboy and this city.

" Resolved also, That a committee be appointed to confer with the health-officer at the Quarantine Ground, and obtain from him, and from such other sources as they shall judge advisable, information of the existence of the said disease, and other facts deemed necessary by them, and report with all convenient speed to this Board.

" Resolved, That the committee appointed be Dr. Joseph Bayley, Dr. John H. Douglass, of this Board, and Dr. David Hosack, of this city; and that the health-officer, Dr. Rodgers, be associated with them.

" *Board of Health, September* 19, 1811.—Present, the Honorable Dewitt Clinton, President.—*Aldermen,* Mesier, Carpenter, Dickinson, Cunningham, Hoogland, Buckmaster, C. Pell, and J. Pell.—*Doctors,* Jones, Miller, Bayley, and Douglass.

" The minutes of the meeting of the 16th were read and approved.

" The committee appointed to confer with the health-officer, and to ascertain the facts respecting the disease prevailing at Amboy, presented the following Report, which was approved, and ordered to be entered on the minutes.

" *Report of the Committee appointed by the Board of Health to investigate the nature and origin of the disease now prevailing at Perth Amboy, New Jersey, addressed to the President and Members of the Board.*

" Gentlemen,

" Agreeably to the instructions conveyed by the resolution of the Board of Health, we yesterday proceeded to Perth-Amboy, for the purpose of obtaining information relative to the nature and origin of the disease, at present prevailing in that city. The health-officer, Dr. Rodgers, having been appointed with us a member of the committee, we waited on him at Staten Island, with the hope that, as he had been at Amboy, and had seen some of those persons who had died of the disease, he would have had it in his power to furnish the information required, and perhaps rendered our visit at Amboy unnecessary; and, in case he should not be in possession of the facts which would be expected by the Board of Health, to request him, as a member of the committee, to accompany us for the purpose of making the necessary inquiries.

"Upon our arrival at the Quarantine Ground, we accordingly stated to the health-officer, that information had been received by the Board of Health, of the prevalence of an infectious disease at Perth-Amboy, which had excited considerable alarm among the citizens of New York. That, in consequence of this information, the mayor had thought it necessary to issue his proclamation, interdicting the communication between the two places, and that the Board of Health had appointed a committee, for the purpose of inquiring into the facts of this subject: that Dr. Millar, the resident physician, had been requested by the Board to be a member of the committee, but, owing to unavoidable engagements, he had declined the appointment. That thereupon they considered it expedient to request the health-officer to perform that duty, and, understanding that he had already been at Amboy, and had expressed an opinion of the nature of the disease, they had been in expectation of receiving from him an official statement of the facts that were now sought for.—Dr. Rodgers replied, "that he did not consider the Board as having any right to expect a communication from him, relative to a fever at Amboy." We then observed, that, as a constant communication existed between Amboy and New York, and that, as the disease might be conveyed to the latter city, they expected from him, as the sentinel at the out-post, every information in his power to communicate, and that, by a resolution of the Board, they certainly did expect that he would also communicate to us the facts that had come to his knowledge.—Dr. Rodgers then stated, "that when this disease might come under his review at the Quarantine Ground, he should consider it his duty to make such communication, but not till then;" and added, "you had better, gentlemen, proceed to Amboy, to seek information for yourselves:" that his duty did not permit him to accompany us; that he should go again to Amboy to-morrow, and pay a visit of friendship, as his two former visits had been, but should not go in an official capacity. We then expressed the hope, that if he did not think it proper to accompany us, that he would favor us with his opinion of the nature of the disease, and the facts relative to its origin. This also he declined, observing, "that he was delicately situated, and did not wish to express to us an opinion on this subject. We then asked him if he had any objection to give us a statement in writing for the information of the Board, and that we would call for it on our return from Amboy. This he also begged leave to decline; adding, however, that, if the Board of Health would do him the honor to address him a letter, stating the points on which they wished information, he would answer their questions. We then read to him the resolution of the Board, requesting us to obtain from the health-officer such information as he possessed on this subject; and added, that we considered the application conveyed by the present resolution, as sufficiently respectful and expressive of the wishes of the Board for him to communicate the information requested; he, however, persisted in declining to express to us a committee from the Board, either verbally or in writing, any opinion on this subject, repeating, "that his situation was a very delicate delicate one." We then observed, that we had understood he had publicly expressed his opinion, at Amboy, of the nature of the fever prevailing there: he acknowledged he had visited Mr. Kearney at Amboy; that he had also seen Mr. Compton; and that he did express to the two physicians of that place, and in presence of a third person, that Mr. Kearney's case was that of "malignant fever of the highest grade."

"As the term *malignant fever* appeared to us of ambiguous import, we next inquired if he considered the fever referred to as the *yellow fever*. To this question he answered, he disliked the term *yellow fever*, considering it an improper one; but admitted that the disease was such as would by many persons be denominated yellow fever; and particularly mentioned, that the patient had the *glassy eye* and *yellow skin*. We next asked him if Mr. Kearney had had the *black vomit;* to this he replied in the affirmative; but that in the other case the stomach was not so much affected. He also stated, that Mr. Kearney had been subject to attacks of *bilious fever*, and that, upon this late occasion, had been very much fatigued, and, prior to his attack, had been exposed to the sun without an umbrella. We repeated our request, that he would accompany us on our visit, but, he again declining to comply with our wishes, we considered our conference at an end.

"We immediately proceeded to Amboy, and called upon Mr. Daniel Perrein, the collector of the port, and Dr. Nathaniel Manning, the physician who had attended on all those who had been attacked with the disease. The other physician being out of town, we had no opportunity of conversing with him. Dr. Manning informed us, that he had lost four patients with a fever, which, from its peculiar symptoms and great mortality, he considered to be the yellow fever; that, although he had been two years and a half a practitioner at Perth-Amboy, and his partner, Dr. Freeman, had practised medicine in the same place many years before him, and both had been familiarly conversant with the fevers which ordinarily prevail in that city and its vicinity, particularly the bilious remittent, yet that they had never before the present season, met with any fever attended with the symptoms of that of which their late patients had died, and with which others at that time were affected; and, as further evidence, Dr. Manning remarked, that at that very time he had patients ill of both diseases, each exhibiting its characteristic symptoms, and invited us to see them. He also stated, that our health-officer, Dr. Rodgers, had visited with him two of the first cases, and had pronounced them as decided cases of the yellow fever as he had ever seen.

"Dr. Manning then gave us an account of those persons who, in his opinion, had died of the yellow fever, namely, James Compton, who was attacked on the 7th, and died on the 10th, instant; James Kearney, attacked on the 9th, and died on the 14th; Joseph Compton, attacked on the 11th, and died on the 15th; and Mrs. Crowell, also attacked on the 11th, and died on the 15th. He observed, that a Mrs. Marsh, an elderly lady, died yesterday morning, the 17th, after a week's illness, but her disease did not appear to be charac-

 terised terised by the same symptoms, that had distinguished the cases of the first persons that have been mentioned. The first four, he remarked, were all seized with severe pain in the head, back, and limbs, attended with a highly inflamed state of the eyes. On the second or third day the skin, especially about the neck and breast, became yellow, which colour gradually extended over the greater part of their bodies; and, in all, the stomach was very much affected with a sense of heat or burning, and rejected almost every thing received into it; which symptoms he had never found associated in the ordinary autumnal, or any other fevers in that place or neighbourhood. We asked him particularly if he had seen Mr. Kearney in any of his former attacks of fever, to which he had been subject in the autumn, as before related to us by Dr. Rodgers. He replied, he had been his physician in two such attacks, and that his disease exhibited the ordinary characters of our common autumnal remittent fever, and was totally different from the inflammatory symptoms with which he was attacked in his last illness. He particularly remarked, that Mr. Kearney threw off a great quantity of black matter, resembling coffee with the grounds, and mixed with other portions in the form of flakes. We next asked Dr. Manning for his opinion relative to the origin of this extraordinary disease. He replied, that there was but one opinion, either with the inhabitants or physicians, namely, that it was derived from some of the West-India vessels, which had been lying at the wharves; observing, however, that the brig Ocean, from St. Bartholomew's, was the vessel which the inhabitants in general supposed to have introduced it. The town we observed to be remarkably elevated; the soil chiefly composed of sand; free from all lodgments of water; the streets wide, and the houses, for the most part, spacious, and at a considerable distance from each other, and the whole town exhibiting an uncommon degree of cleanliness. Dr. Manning also informed us, that there were no local causes to which this calamity could possibly be referred, and that it could only be accounted for as arising from intercourse with the vessels at the wharves, and that the citizens were so perfectly convinced of this fact, that they had ordered them to be removed to the stream. He further stated, that Mr. Kearney, and the two Mr. Comptons, had all been frequently on-board the brig Ocean, and another vessel lying alongside of her, the ship Favorite, from the Havanna, both of which vessels had come consigned to Mr. Kearney. He also observed, that Mr. Joseph Compton had not only been on-board of the vessels at the wharves, but that he had sat up with his brother James, during his illness.

" Mrs. Crowell, the fourth person mentioned, was the wife of a ship-carpenter, who resides at the head of the wharf, within 50 yards of the said vessels. Dr. Manning also stated, that Mrs. Crowell was so near to the vessels, that, upon one of them (the Ocean) having her bilge-water pumped out, she was made very sick by the smell of it, the wind blowing directly towards the house, and into her apartments; and that she herself, during her illness, frequently declared to her physician and friends, that in her opinion she had thus taken the disease. It will be readily observed, that the same wind which blew the effluvia of the bilge-water, would also convey the poisonous vapor from the adjoining vessel.

"Having received this information from Dr. Manning, we accompanied him in his visit to five of his patients whom he considered to be the most dangerously ill. We united with him in opinion, in pronouncing three of those, viz. Miss Ann Taylor, Captain James Baynon, and Mr. Voorhees, to be *decided cases of yellow fever*. The diseases of two others, Mr. Semple, and Mr. Kane, were readily distinguished as the *common bilious remittent of autumn*.

"The alarming situation of Miss Taylor particularly arrested our attention, although this was the second day of her illness; her symptoms (so peculiar are the features of this disease) were of a character that declared her to be in the most imminent danger; and with all the kind assistance she receives from her amiable sisters, and the skill of an attentive physician, we have too much reason to fear that she must soon be numbered among the victims of this deadly disease. Miss Taylor resides some distance from the source of infection, but, on inquiry, we found that she had been unfortunately in the neighbourhood of the vessels, by frequently visiting a friend in the house adjoining to that in which Mrs. Crowell lay ill and died, and that too at the very time of Mrs. Crowell's illness. We learned also, that the other persons taken ill, had been exposed either directly by being on-board the vessels, or by visiting those who were ill of the disease. Many other persons, Dr. Manning informs us, had been just seized with fever, but so recently, that he would not yet undertake to pronounce on the nature of the disease.

"Our attention was also directed to the vessels supposed to have introduced the fever. For this purpose, we applied to the collector of the port, who furnished us with the following facts. That twelve vessels had arrived at that port from the West Indies since the first day of June. That many others had arrived from Ireland, full of passengers, in a most filthy condition, but remarkably healthy. He then informed us, that the brig Ocean, the vessel at first suspected, arrived at the wharf on the 4th of September, from Bartholomew's; that she was laden with rum, sugar, molasses, and coffee; that four or five days after her arrival she began to discharge her cargo. The master, Capt. Sutton, being in New York, we had no opportunity of obtaining any information from him. The collector stated, that Mr. Kearney, as the consignee, was frequently in the hold of the vessel. Capt. Little, a passenger in the Ocean, informed us, that her crew had been healthy, but admitted that her bilge-water was in a very offensive state, and that, in this respect, she was a filthy vessel, but not more so than West-India traders in general.

"Mr. Perrein next stated, that the ship Favorite, James Stuart, master, the other suspected vessel, arrived from the Havanna, and came to the dock on the 30th of August, and was also consigned to the late Mr. Kearney; and that she, with the other vessels, was removed to the stream on Sunday last: he added, that Mr. Kearney, the consignee, and the two Mr. Comptons, had been also frequently on-board of this vessel. Capt. Stuart being in Amboy, we availed ourselves of the opportunity of conversing with him, relative to the state state of his vessel. He, with great frankness, gave us every information we requested. He stated, that he first went to North Carolina, from Perth-Amboy; that he next proceeded to Falmouth, (Jamaica,) and from thence to the Havanna, and that he had brought the crew all back with him in a state of health; that they had all been several voyages to the Havanna, some three, four, and five, voyages, and were all well-seasoned to the West-India climate: he also acknowledged, *that it was very sickly at the Havanna when he was there.* Upon questioning him relative to his vessel, he also very candidly stated, that his ballast had not been shifted from the time he left Amboy to this day. Such are the facts we have been enabled to obtain on this subject, and which we beg leave to present to the Board without delay.

"As it has been made our duty to express an opinion of the nature and origin of the disease in question, we feel no hesitation in saying, first, that the disease, in our opinion, is that commonly known and described as the *Yellow Fever;* and, secondly, that it has been satisfactorily traced to one of the two last-mentioned vessels arriving from the West Indies, and that it has most probably been derived from the *ship Favorite, from the Havanna*, which is by all acknowledged to have been a sickly port at the time of her sailing. Nor do we conceive it to be an objection to the correctness of this opinion, that the crew remained healthy throughout the voyage, as it is also stated, that, by the several voyages they had made to the Havanna, they had become seasoned to that climate.

"It will be readily recollected by the Board, that a similar instance occurred in July 1809, at which time two persons from this city, worked on-board the brig Mary, a vessel from the Havanna, then detained at the Quarantine Ground, and were seized with the yellow fever, of which they died in this city, although the crew of the same vessel had been in perfect health during the whole of the voyage.

"We cannot conclude this communication without remarking, that Mr. Perrein, the collector at Amboy, and Dr. Manning, a physician of the same city, are entitled to our acknowledgments for the very unreserved and candid manner with which they communicated to us the information they possessed relative to the subject of our inquiries. "We are, Gentlemen, very respectfully,

"Your humble Servants,

"DAVID HOSACK,
"JOS. BAYLEY,
"JOHN H. DOUGLASS.

"*New York, September* 18, 1811.

"*To the President and Members of the Board of Health.*

"Extracted from the Minutes. J. MORTON, Secretary."

"The Board of Health of Philadelphia have issued a proclamation, prohibiting all intercourse and communication between the city of Amboy, and the city and county of Philadelphia, on account of a pestilential disease prevailing in the former city. Persons, after leaving Amboy, must perform fourteen days' quarantine before they can be admitted into Philadelphia."

(To be continued.)

Medico-

*Medico-Chirurgical Transactions, published by the Medical and Chirurgical Society of London.* Vol. II. 8vo. pp. 420. plates. Longman and Co. 1811.

(Continued from p. 68.)

A case of dysphagia, produced by aneurism of the aorta, related by Mr. Armiger, affords considerable interest. The patient complained that whatever he swallowed seemed to stop in his breast; that he felt great uneasiness just below the margin of the ribs on the left side; and that he seldom obtained relief, till, by frequent efforts, he succeeded in expelling the solid matter which would not pass downwards. Stricture in the œsophagus was immediately suspected; but, on examining the seat of uneasiness, and pressing on the epigastric region, Mr. Armiger could not discover either internal enlargement or hardness. The man had been subject to frequent palpitations of the heart; and, having walked some distance previous to Mr. Armiger seeing him, it beat at the time more violently than usual.

"On applying one hand to his left side, and the other to the sternum, I readily distinguished (says Mr. A.) the agitated action of the heart, from a pulsation, attended with a sensation which is occasioned by blood passing into an aneurismal tumor. I conjectured that some part of the descending aorta was the seat of aneurism, and that the dysphagia was occasioned by the pressure of the tumor on the œsophagus.

"He was now much emaciated, and his strength was much impaired; he stooped considerably, and in that posture, while leaning on his stick, or other firm support, was easier than when erect; more so when lying in bed, but most of all while resting on his knees and elbows. He lay with difficulty and pain even for a few minutes on his back. When on his left side, his respiration was hurried; but at other times, or in other postures, unless from exertion, it was scarcely affected. When the pulsation was most violent, it might readily be felt between his shoulders: the pain was at times piercing, shifting from his breast to his back, and *vice versa.* When in the back, it was principally seated immediately below the inferior angle of the left scapula."

His difficulty in swallowing first occurred in December 1809. His debility and other symptoms increased till the morning of the 2d of April, 1810, when he vomited up nearly a pint of blood. In the afternoon his pulse intermitted considerably, and the pulsation of the aneurism was much reduced in strength. In the evening he lost, at once, a quart of blood, fainted, and expired.

Dissection presented the following appearances:

"*Abdomen.* In this cavity there were four ounces of fluid, deeply tinged with the red particles of the blood. The stomach, duodenum, jejunum, jejunum, and transverse colon, were considerably distended with flatus; the other parts of the intestinal canal were unnaturally contracted. The pyloric extremity of the stomach, the beginning of the duodenum, part of the jejunum and of the ilium, with the cœcum, were of a reddish purple color, occasioned by an effusion of fluid between the peritoneal and muscular coats of those organs, which extended, in some places, between the layers of the mesentery and mesocolon.

" *Thorax.* In both the cavities of the chest there was a fluid effused similar to that in the abdomen, in quantity amounting to about seven ounces in each cavity. The surface of the lungs was discolored like that of the intestines, and from the same cause.

" *Pericardium.* In the cavity of this membrane there were four ounces of fluid, of a darker color than that either in the abdomen or thorax.

" The heart, pulmonary artery, and the aorta, nearly as far as the seat of the aneurism, exhibited the natural appearances.

" On raising the lungs, which were healthy, the aneurismal tumor, bounded by the layers of the posterior mediastinum, was readily distinguished. It was in the descending aorta, and was situated on the 8th, 9th, 10th, and 11th, dorsal vertebræ; but they had not, in the least degree, suffered from its presence. It seemed to be capable of containing about a pint of blood. The layers of the mediastinum, the side of the œsophagus against which it pressed, and the cellular membrane connecting the parts in the cavity of the mediastinum, formed part of the parietes of the tumor. Within it, and adhering to its inner surface, and to each other, were several concentric strata of coagulated blood. Towards the upper and right side, the coats of the aorta were much thinner than in any other part, excepting immediately contiguous to the spot at which it finally burst into the œsophagus, at about two inches distant from the passage of that tube through the diaphragm.

" The tumor seems to have been primarily formed by dilatation of the aorta, the larger part of it being on that side of the aorta which is contiguous to the œsophagus. It was probably enlarging principally on its right side, during the time that the remission of the dysphagic symptoms occurred. A little beyond the root of the left subclavian artery, there was also a small aneurismal dilatation."

Mr. Astley Cooper has contributed the dissection of a limb on which the operation for popliteal aneurism had been performed seven years. Besides demonstrating the form and course of the new circulation, the paper is valuable from the detail of some experiments made upon dogs; by which it appears, that one of the animals lived " more than twelve months with the *two carotids*, *the two femorals*, *and one brachial artery*, *obliterated*;" and another survived the operation of tying and dividing the aorta, maintaining his usual health, and the ligatures coming away as other ligatures upon upon arteries. The anastomosing vessels were large and numerous.

Mr. Cooper has also communicated some cases of Spina bifida, which were treated upon a new plan, and with signal success. We know of no discovery in our art, since that of vaccination, that has such a high claim upon our gratitude and admiration. Infants, which before were doomed to certain destruction, with scarcely an attempt at relief, may now, in many instances, be preserved to their friends, and become valuable lives to the community.

For the relief of this complaint, Mr. Cooper proposes two modes of treatment; the one palliative only, the other radical.

"The first consists in treating the case as a hernia, and applying a truss to prevent its descent; and the second in producing adhesion of the sides of the sac, so as to close the opening from the spine, and stop the disease altogether. The first is attended with no risk. The truss forms an artificial vertebra, when the natural is defective, a buttress which supports the part, and prevents the increase of the disease; but in this mode of treatment, the truss is required in future life, for if discontinued the tumor re-appears, and will grow, as hernia does, to great magnitude, but with more fatal consequences. On the contrary, the adhesive mode of cure exposes the patient to much constitutional irritation, but leaves him without the apprehension of the future return of the disease."

If this mode should fail, Mr. Cooper remarks that it does not prevent our subsequently attempting the palliative treatment.

There are some cases of Spina bifida, however, which cannot be cured.

"If the tumor is connected with an unnatural enlargement of the head, hydrocephalus internus is conjoined with spina bifida; and the water will accumulate in the ventricles, if the tumor in the loins is attempted either to be palliated or radically cured. If the lower extremities are paralytic, or the fœces and urine are discharged involuntarily, there is no hope of relief.

"If the tumor has burst at the time of birth, or bursts soon after, there is little hope of cure; for, although the opening in the skin may be closed by lint and adhesive plaister, and union be produced so as to admit of no further discharge of water, yet hydrocephalus internus will still succeed.

"The deficiency of the spine is sometimes so great as to lead to the production of a most extended tumor at the time of birth, and when this is the case, the nerves are so far protruded from the spinal canal as to injure the structure of the spinal marrow, and to render every attempt at cure unavailing."

Mr. Cooper continues,

"I should feel myself deficient in that liberality with which our profession profession ought ever to be marked, and usurping more than my due, if I did not state that the principle of the radical cure, as proposed for Spina bifida, is similar to that recommended by Mr. Abernethy in his work on psoas abscess. The mode, however, which I have employed for the purpose is, I believe, the only safe one, that of puncturing the part with a needle; for every opening of a larger size will be attended with the utmost danger.

"I have, for many years, used this plan in cases of ganglia, when I could not burst them by a blow, or excite their removal by pressure or irritation; and I have never seen it followed by inflammation or any serious consequence; and it may be used in cases of accumulations in joints, and other cavities, where larger openings are dangerous."

A fatal case of Hydatid in the substance of the brain, is related by Mr. Morrat, of Worthing. It occurred in a robust young woman, whose general health was good. The symptoms which indicated the seat of the complaint were vertigo; disordered stomach; ferrety appearance about the eyes; fixed pain in the head; and fits. These increased till she lost her sight, hearing, and smell. She became paralytic, fell into apoplectic stupor, and died. Except the hydatid, and encreased fulness of vessels, there was no diseased appearance in the brain.

Mr. Park, of Liverpool, has contributed some interesting cases and observations on Tumors within the Pelvis occasioning difficult parturition.

Mr. P. T. Creagh, of the royal navy, has given the particulars of a case of fractured cranium, where the dura and pia mater were lacerated, and a great quantity of the cerebrum protroded. The patient recovered, although a considerable portion of brain was lost.

Mr. Lawrence, of St. Bartholomew's hospital, has related the particulars of a very curious case, in which the patient, a young woman, was afflicted with a peculiar undescribed species of worms in the bladder. The symptoms produced were highly distressing; and, before the existence of worms was ascertained, she was sounded on suspicion of calculus. Oil of turpentine injected into the bladder, had the effect of removing these troublesome insects, a representation of which is given in a plate. We had great pleasure in perusing the narrative, from the clear and interesting style which Mr. Lawrence has happily succeeded in obtaining.

A successful case of Trismus, following a contused wound in the head, in a laboring man, is communicated by Mr. Harkness, surgeon, at Ratcliffe. Mr. Thomas Blizard, of the London Hospital, also attended the patient; and, as the treatment was bold, and the result happy, we shall extract the the Table which the author has presented, exhibiting the daily amount of each remedy.

TABLE.

| | Tinct. Opii. | Calo-mel. | Jalap. | Ext. Col. | Gam-boge. | Scam. | Wine. | Porter. |
|---|---|---|---|---|---|---|---|---|
| | dr. | gr. | dr. | gr. | gr. | gr. | Bottles. | Pints. |
| February 26 | 8 | 25 | 2½ | | | 15 | 3 | |
| 27 | 8 | 32 | 4 | | | | 3 | 2 |
| 28 | 12 | 48 | 6 | | | | 3 | 6 |
| 29 | 5 | 20 | 1 | | | | 3 | 5 |
| March 1 | 6 | 35 | | 36 | 20 | | 3 | 7 |
| 2 | 7 | 35 | | 45 | 35 | | 2 | 1 |
| 3 | 8 | 40 | | 40 | 40 | | 1 | 3 |
| 4 | 10 | 50 | | 48 | 50 | | 1 | 3 |
| 5 | 8 | 40 | | 48 | 40 | | 2 | 8 |
| 6 | 8 | 40 | | 48 | 40 | | 2 | 8 |
| 7 | 8 | 40 | | 48 | 40 | | 2 | 8 |
| 8 | 8 | 40 | | 48 | 40 | | 2 | 8 |
| 9 | 8 | 40 | | 48 | 40 | | 2 | 7 |
| 10 | 8 | | | 80 | 40 | | 2 | 6 |
| 11 | 8 | | | 80 | 40 | | 2 | 7 |
| 12 | 8 | | | 80 | 40 | | 2 | 7 |
| 13 | 8 | | | 80 | 40 | | 2 | 8 |
| 14 | 8 | | | 80 | 40 | | 2 | 8 |
| 15 | 8 | | | 80 | 40 | | 2 | 8 |
| 16 | 8 | | | 80 | 40 | | 2 | 8 |
| 17 | 8 | | | 80 | 40 | | 2 | 8 |
| 18 | 8 | | | 80 | 40 | | 2 | 8 |
| 19 | 4 | | | 40 | 20 | | 2 | 8 |
| 20 | 4 | | | 40 | 20 | | 1½ | 9 |
| 21 | 4 | | | 40 | 20 | | 1½ | 10 |
| 22 | 4 | | | 40 | 20 | | 1½ | 10 |
| 23 | 4 | | | 40 | 20 | | 1½ | 10 |
| 24 | 8 | | | 40 | 20 | | 1 | 10 |

A second case of Trismus, occurring in a female, in consequence of a compound fracture of the leg, and treated in nearly a similar manner, by Sir William Blizard and Mr. Parkinson, jun. of Hoxton, also succeeded well. The patient, at one time, took a dram of tincture of opium every hour for several days successively, without the sensorium being affected.

In a letter to Mr. Chevalier, who in his lectures had recommended large doses of arsenic after the bites of venomous animals, Mr. Ireland, army-surgeon, states, that he had some opportunities of witnessing the good effects of that remedy in the course of his practice in the West Indies.

In some of the islands, venomous serpents abound; in St. Lucia is found the coluber carinatus, (Linn.) It is from

 three three to six feet in length, with fangs from one and a half to two inches long; the wound it inflicts is generally of considerable extent, and the bite is deadly.

On Mr. Ireland's arrival in the island, he was informed that an officer and several men, belonging to the 68th regiment (to which he was attached) had recently died from the bites of those destructive reptiles, within six and twelve hours from the time of receiving the wound, notwithstanding every effort that had been made by the medical gentlemen present.

A case soon came under the author's immediate care, which, from its importance, we shall state in his own words:

"Jacob Course, soldier, in the York light infantry volunteers, was bitten in the left hand, and the middle finger was so much lacerated, that I found it necessary to amputate it immediately, at the joint with the metacarpal bone.

"I first saw him about ten minutes after he had received the wound, and found him in a torpid, senseless, state: the hand, arm, and breast, of the same side, were much swelled, mottled, and of a dark purple and livid color. He was vomiting, and appeared as if much intoxicated. Pulse quick and hard: he felt little or no pain during the operation.

"The wound being dressed, and the patient put to bed, I ordered a cathartic glyster, and the following medicine to be taken immediately: ℞. Liq. Arsenic. ʒij. Tinct. Opii gtt. x. Aq. Menth. Pip. ℥iſs. which was added to half an ounce of lime-juice; and, as it produced a slight effervescence, it was given in that state. This remained on his stomach, and was repeated every half hour for four successive hours. In the mean time the parts were frequently fomented with common fomentation, and rubbed with a liniment composed of Ol. Terebinth. ℥ſs. Liq. Ammon. ℥ſs. and Ol. Oliv. ℥iſs. The cathartic glyster was repeated twice when the patient began to be purged; the arsenical medicine was now discontinued. He had become more sensible when touched, and from that time he gradually recovered his faculties; he took some nourishment, and had several hours' sleep.

"The next day he appeared very weak and fatigued; the fomentation and liniment were repeated. The swelling diminished gradually; the natural color and feeling returned, and, by proper dressings to the wound, and attention to the state of his bowels, he soon recovered, and returned to his duty."

Some other cases are stated in which arsenic was given in large doses with similar good effects. The paper concludes with a note from Mr. Chevalier, in which he states, that he was induced to recommend arsenic in such cases from the facts recorded in Dr. Russell's History of Indian Serpents, on the authorities of Mr. Duffen and Mr. Ramsay.

We

We have now gone through the most important articles in the volume; the copiousness of our extracts may indicate that we deem this growing collection of facts, and remarks, useful to the profession, and tending to improve the arts of medicine and surgery.

